# Firth’s Logistic Regression of Interruption in Treatment before and after the Onset of COVID-19 among People Living with HIV on ART in Two Provinces of DRC

**DOI:** 10.3390/healthcare10081516

**Published:** 2022-08-12

**Authors:** Gulzar H. Shah, Gina D. Etheredge, Jessica S. Schwind, Lievain Maluantesa, Kristie C. Waterfield, Astrid Mulenga, Osaremhen Ikhile, Elodie Engetele, Elizabeth Ayangunna

**Affiliations:** 1 Department of Health Policy and Community Health, Jiann-Ping Hsu College of Public Health, Georgia Southern University, Statesboro, GA 30460, USA; 2 FHI 360, Washington, DC 20001, USA; 3 FHI 360, Kinshasa 1015, Democratic Republic of Congo

**Keywords:** people living with HIV, treatment interruptions, antiretroviral therapy (ART), Democratic Republic of Congo, Firth’s logistic regression, COVID-19

## Abstract

The impact of the COVID-19 pandemic extends beyond the immediate physical effects of the virus, including service adjustments for people living with the human immunodeficiency virus (PLHIV) on antiretroviral therapy (ART). Purpose: To compare treatment interruptions in the year immediately pre-COVID-19 and after the onset of COVID-19 (10 April 2020 to 30 March 2021). Methods: We analyze quantitative data covering 36,585 persons with HIV who initiated antiretroviral treatment (ART) between 1 April 2019 and 30 March 2021 at 313 HIV/AIDS care clinics in the Haut-Katanga and Kinshasa provinces of the Democratic Republic of Congo (DRC), using Firth’s logistic regression. Results: Treatment interruption occurs in 0.9% of clients and tuberculosis (TB) is detected in 1.1% of clients. The odds of treatment interruption are significantly higher (adjusted odds ratio: 12.5; 95% confidence interval, CI (8.5–18.3)) in the pre-COVID-19 period compared to during COVID-19. The odds of treatment interruption are also higher for clients with TB, those receiving ART at urban clinics, those younger than 15 years old, and female clients (*p* < 0.05). Conclusions: The clients receiving ART from HIV clinics in two provinces of DRC had a lower risk of treatment interruption during COVID-19 than the year before COVID-19, attributable to program adjustments.

## 1. Introduction

Sustained care and antiretroviral therapy (ART) are imperative for increased survival time among people living with HIV (PLHIV) and constitute a crucial component in preventing community spread [1]. In the Democratic Republic of Congo (DRC), where the HIV incidence rate per 1000 individuals for all ages is 0.18 with a 95% CI [0.10–0.30], limited research explored the impact of retention rates (also known as “continuity of treatment”) on people living with HIV [2]. According to a prospective cohort study conducted in Kinshasa, rates of continuity of treatment (CoT) between 2016 and 2018 were 50% and 21% at 6- and 12-month follow-up appointments, respectively [3]. The characteristics positively associated with CoT were low economic status, enrollment in an educational institution, Internet access, previous HIV tests, and having plans to disclose positive HIV tests to partners [4,5]. CoT was negatively associated with depressive symptoms, distance to an ART center, and a lack of disclosure [4,5]. A retrospective analysis of ART-naive women aged 15–45 years initiating HIV care at two large clinics during 2007–2013 showed that HIV-infected women that were pregnant during care enrollment were more likely to never return for follow-up [6].

Variation in treatment interruption (also known as loss to follow-up (LTFU)) was observed across varying characteristics in the DRC [3,7]. In Haut-Katanga, a region known for its large copper and cobalt mines [8], the odds of LTFU were significantly lower for patients in this region compared to those in Kinshasa, the capital city [9]. Additionally, infants in Kinshasa whose mothers were not on ART were more than twice as likely to experience treatment interruption compared to those who were on ART [10]. The odds of experiencing treatment interruption were the lowest among patients with the World Health Organization (WHO) clinical stages 1 or 2 when compared to those in stages 3 or 4 [11]. To improve CoT, measures such as continuous quality improvement [12] and conditional cash transfers were suggested [13].

Effective strategies adapted to local contexts that directly address factors affecting retention, LTFU, and adherence to care and treatment, engender substantial benefits for PLHIV on ART [14]. Studies have shown that, before the COVID-19 pandemic, efforts to reduce the burden on the healthcare system while ensuring CoT, such as through the differentiated care model (DCM), [15,16] resulted in similar CoT rates and was an acceptable alternative to standard HIV care for PLHIV [17]. DCM and the resulting differentiated service delivery is a client-centered approach that simplifies and adapts HIV services across the cascade to reflect the preferences and expectations of various groups of PLHIV while reducing unnecessary burdens on the health system. In recent studies with similar CoT rates in sub-Saharan countries, this model employed strategies, such as facility fast-track drug refills, appointment spacing, community or home-based care, and decentralization of care [17,18,19,20,21]. Similar care models have been proposed during COVID-19 to enhance continuity of care for PLHIV in the DRC. In March 2020, the WHO recommended less frequent clinic visits and HIV services and programs to adopt multi-month dispensing (MMD) for PLHIV who were stable (defined as someone older than two years, on ART for 12 months, without active opportunistic infections (OI), and with a suppressed viral load (VL) (including females that are pregnant or breastfeeding)) [22]. In concert with these guidelines, the President’s Emergency Plan for AIDS Relief (PEPFAR), in congruence with advocacy by the International AIDS Society [23,24], placed special emphasis on CoT for PLHIV through the expanded use of decentralized and MMD of ART. Furthermore, the decentralized distribution models specific to the COVID-19 management of HIV included community pharmacy, automated dispensing models, and private hospital models [23,25].

The potential effects of disruption to HIV programs and CoT in sub-Saharan Africa caused by COVID-19 were forecasted to result in increases in mortality risk, most notably due to the disruption to the supply of ART resulting in an individual’s discontinuation of ART [26]. In addition to a six-month interruption of ART supply, other disruption scenarios included reduced outreach and access to clinics, which was expected to cause a decrease in ART initiations, HIV testing, and the proportion of PLHIV that are VL suppressed [27]. However, a recent study that tried to understand the impact of the pandemic on HIV treatment in Sub-Saharan countries, among which was the DRC, indicated the overall number of people receiving ART did not decline during the year, but rather steadily rose [28]. According to this study, which occurred in 1058 facilities across 11 Sub-Saharan countries, there was an initial decline in the number of people tested for HIV (January–June 2020). The observed decline was followed by an increase in the number of people who initiated ART, received viral load (VL) testing, and were VL suppressed [28].

As COVID-19 is still ongoing in the DRC, there is a paucity of studies investigating CoT rates during COVID-19. Hence, the purpose of this study was to fill that gap to determine the CoT rate of people living with HIV in the Democratic Republic of Congo, in particular Kinshasa and Haut Katanga provinces, prior to and during the COVID-19 epidemic.

## 2. Materials and Methods

### 2.1. Data

Our study used a retrospective cohort design based on the date of ART initiation reported in data collected for routine HIV program administration. The study setting included 313 HIV care clinics in the Haut-Katanga and Kinshasa provinces of the Democratic Republic of Congo. The study sample was limited to 313 clinics (in 29 health zones) owned by the DRC government, as well as private and faith-based organizations that are supported by the implementing partners participating in the National HIV/AIDS Program (PNLS), and are supported by the Centers for Disease Control and Prevention (CDC) through PEPFAR. Data were obtained for this study from the HIV program implementing partners, SANRU, HPP, and ICAP in April 2021, covering the 36,585 persons with HIV who initiated ART between 1 April 2019 and 30 March 2021. These data were gathered by the implementing partners using a clinical database to track HIV counseling, testing, and service delivery encounters using an electronic patient management system (TIER.Net Version 1.10, Cape Town University, South Africa). For this research, only a limited number of de-identified data elements were made available. Georgia Southern University’s Institutional Review Board (IRB) approved the study (Protocol no. H-l9260).

### 2.2. Dependent Variable

The dependent variable treatment interruption was operationalized based on the data field noting the patient outcome. The program staff coded the clinical outcomes as one of the four attributes recorded for each of the visits—(a) died, (b) in care, (c) transferred out, or (d) lost to follow-up (LTFU). The HIV clinics marked the outcome as LTFU to document interruption in the treatment that had occurred when a person living with HIV who was on ART had a lapse of 180 days or longer since the last clinic visit or expected date of return. After excluding the deceased clients, the variable treatment interruption was coded as “1” for outcome was LTFU, and coded as “0” for attributes transferred out to another facility or in care.

### 2.3. Independent Variables

The primary independent variable, whether ART initiation occurred during COVID-19, was coded as “pre-COVID-19” if ART initiation started in the year immediately pre-COVID-19 (1 April 2019 to 9 Apri1 2020) and “during-COVID-19” if the ART was initiated on or after the onset of COVID-19 (10 April 2020 to 30 March 2021). The demographic characteristics selected as covariates included the client’s sex (female or male) and age at the time of the last visit (15 years or older vs. <15 years of age), which is the standard PEPFAR disaggregating and reporting norm. The urban/rural status of the health zone was coded as rural or urban. The clinical variables included duration on ART in months (continuous variable) and TB status on the last visit (no TB symptoms detected vs. TB detected based on symptoms, clinical diagnosis, or treatment). Our selection of the covariates was extremely limited because we only had access to the deidentified, restricted dataset. Additional contextual variables representing community-level social determinants of health were desirable but unavailable.

### 2.4. Analytical Methods

Descriptive statistics, such as frequency distribution, percentages, and arithmetic means for all independent and dependent variables, were calculated to provide contextual information about the study participants’ clinical and demographic characteristics. To test the bivariate associations between each of the categorical independent variables and the dichotomous dependent variable, a chi-squared test was used; a t-test was used for the association between the dependent and continuous independent variables. To model the binary outcome interruption during treatment, which was a rare event (particularly during COVID-19), Firth’s logistic regression was used to reduce the bias in maximum likelihood estimates of the coefficients. The use of Firth’s logistic method is useful in reducing the bias when there is a strong imbalance in the outcome, as in our outcome variable [29]. The model selection was performed based on the theory, rather than through one of the variable selection options available in Stata. With the penalized log likelihood of −1661, the McKelvey and Zavoina R2 was 0.35. The association between the independent and dependent variables was considered significant when *p* ≤ 0.05. All analyses for this study were performed using Stata Statistical Software: Release 16. College Station, TX: StataCorp LLC [30].

## 3. Results

Descriptive statistics for people with HIV who initiated ART between 1 April 2019 and 30 March 2021 indicated that 52.5% initiated ART in the pre-COVID-19 period from 1 March 2019 to 9 April 2020, and 47.5% initiated ART during the COVID-19 period from 10 April 2020 to 30 March 2021 (Table 1).

Treatment interruption occurred in 0.9% of clients with a CoT rate of 99.1%. A small percentage of people living with HIV (1.1%) had TB, whereas TB was not detected in 98.9%. The average duration of ART for clients included in the study sample was 9.5 months, with a standard deviation of 6.3 months. Descriptive statistics about geographic location and demographic characteristics showed that 74.5% of clients received treatment from clinics located in urban health zones, 60.9% were male, 7.4% were younger than 15 years, and 92.6% were 15 years or older.

The percentage of clients with treatment interruption was significantly higher pre- COVID-19 than during COVID-19, respectively 1.5% and 0.2% (Table 1). The risk of interruption was higher for clients with TB present than those with no TB (4.4% vs. 0.0%), those receiving ART at urban clinics than rural (4.4% vs. 0.9%), younger than 15 years old (1.4% vs. 0.9%), and for females than males (1.1% vs. 0.7%).

Firth’s logistic regression of treatment interruption showed the odds of interruptions in treatment were 12.46 times higher if the clients started ART before COVID-19 than a year after the onset of COVID-19 (adjusted odds ratios or AOR, 12.5; 95% CI, 8.5–18.3), after controlling for all covariates in the model (Table 2).

The odds of treatment interruption were greater for clients with TB present than those with no TB (AOR = 5.1; 95% CI, 3.0–8.5), those receiving ART at urban clinics than rural (AOR, 6.5; 95% CI, 3.9–10.9), those younger than 15 years old (AOR, 1.8; 95% CI, 1.3–2.5), and for females than males (AOR, 1.5; 95% CI, 1.2–1.8).

## 4. Discussion

In this era of continuous quality improvement, creating empirical evidence about the factors associated with treatment interruption is critical to support evidence-based decision making for HIV care programs. It is also important to know what effect, if any, COVID-19-related interruptions and program adjustments had on treatment interruptions, which was the focus of this research. Using a retrospective cohort design, this study analyzed data from 313 HIV care clinics in the Haut-Katanga and Kinshasa provinces of DRC. Overall, the treatment interruption was observed in 0.9% of the people living with HIV and who were on ART during a roughly two-year study period. Against our expectations, treatment interruptions were significantly higher in pre-COVID-19 than during COVID-19, making the adjusted odds of interruptions in treatment 12 times greater for clients starting ART before COVID-19 than in the year after the onset of COVID-19. This may be attributable to no restrictions in movement during the pre-COVID19 era. Hence, during pre-COVID-19 period, clients may have traveled for pleasure or work and missed their appointments. During the COVID-19 era, with movement restrictions in place, clients were likely to stay closer to home and their designated facilities. With the increased focus across the nation on health, during this period they likely prioritized their health over other activities. As noted earlier, in DRC, treatment is considered interrupted upon observing the ART lapse of 180 days or longer since the last clinic visit or expected date of return, which is a long timeframe. Given that the Office of the U.S. Global AIDS Coordinator and Health Diplomacy recommends a shorter timeframe, many PEPFAR programs are trying to make national governments reduce long periods and customize the treatment interruption definitions that are culturally appropriate and properly scaled by age and sex [2]. Studies in other settings, though not comparable, provide some comparisons to our findings. A study of PLHIV in 31 provinces of China used survey data to show that social disruptions during COVID-19 contributed to interruptions in ART interruptions [31]. Other studies in other parts of the world showed that subjects were able to maintain ART during COVID-19 [32,33].

The MMD may have reduced the probability of actual treatment Interruptions, as well as documentation of interruptions in our research. In DRC, the differentiated service delivery approach was adopted before COVID-19, including the Option B+ program implemented by the DRC Ministry of Health in 2013 that provided lifelong ART for all HIV-positive pregnant and breastfeeding women, regardless of the immunological or clinical stage, because it was shown to reduce early loss to retention in care [20]. During COVID-19, MMD was extended to all clients on ART, regardless of their stability status. The WHO’s March 2019 recommendation to reduce the frequency of clinic visits for HIV services through MMD or ART for stable people living with HIV, along with other recommendations made by international organizations, [23,24] was adopted in DRC as well. For effective COVID-19 management of HIV in DRC, MMD was commenced for those aged 2 to 13 years for 3 months (MMD-3) old, as well as MMD for individuals aged 14 years and older for 6 months (MMD-6) during the COVID-19 pandemic. The DRC government also took precautions to avoid supply stock shortages despite shutdown activities and border restrictions. For example, the movement of residents was not restricted within each province, but was restricted between provinces [25,26]. Other studies have shown positive outcomes of MMD because it reduced barriers to access to ART facilities (such as transportation and time off from work) and prevented the overcrowding of clinics, thus reducing the risk of COVID-19 transmission as well [34,35].

This study’s findings should be interpreted within the context of the limitations inherent in research that is based on the analysis of secondary data, originally collected for a different purpose. In this case, the data were collected primarily for HIV services and program administration. First, several contextual variables were not available in the secondary data, the availability of which could have increased the prediction power of the independent variables in the model [31]. For instance, treatment interruption can be influenced by an array of socioeconomic and lifestyle factors not available for our study. Secondly, our study sample was restricted to the 313 HIV clinics supported by the CDC through the PEPFAR program. Therefore, the other HIV service outlets outside of this program were not included. For the same reason, the other geographic areas in DRC could not be covered in the sample. Finally, although the statistical model adjusted for the duration of ART when testing the association between the dependent variable and whether the ART was initiated during COVID-19 (vs. pre-COVID-19), it may be argued that results may still be subject to some bias. Given that it takes 180 days for someone to miss ART and get marked as treatment interruption, the time for risk of ART interruption for those initiating ART in the pre-COVID-19 year was much longer, up to 18 months, whereas that period for those initiating ART during COVID-19 was up to 6 months. Regardless of these limitations, the natural experiment created by COVID-19 and the large-scale robust dataset from HIV clinical services providers in two provinces allowed our findings to fill an important gap in the existing body of research about HIV services in DRC.

Our study results also show the greater odds of treatment interruptions when TB-HIV co-infections existed compared to when TB was not a co-infection, which was in line with the result of a study conducted in Nigeria [35]. TB-HIV co-infection is always a strong risk factor for LTFU. It is probable that the travel restrictions, making persons stay close to home, did not have a differential effect on this population as it may have had on those patients infected solely with HIV, as those co-infected would be less likely than others to travel during normal times. It is also possible that co-infected patients were more likely to get COVID-19, and would be less able to attend clinic appointments or get their ART, making them more likely to be LTFU during this time period. Our study also showed that the risk of treatment interruptions was much greater for clients of urban clinics, perhaps due to a higher likelihood of sicker patients seeking care in urban clinics. Although the definition of treatment interruption differed compared to our study, Dorward and colleagues’ study [33] in South Africa showed that, in the rural clinics, the increase in missed visits during the first week of lockdown was much less marked (IRR 1·274, 95% CI 1.076–1.509) than in the urban clinics. The current study showed higher odds of ART interruptions for clients aged < 15 years, perhaps attributable to the need of the clients < 15 years of age to be accompanied, which may have been harder during the lockdown.

## 5. Conclusions

While some research showed interruptions in HIV services, such as testing during COVID-19, the ART services were, for the most part, maintained at the pre-COVID-19 levels during COVID-19. The current study showed that the risk of treatment interruptions in people living with HIV was much higher in the 12 months before COVID-19 onset than during the 12 months after the onset of COVID-19. The overall level of treatment interruption during the study period of two years was also very low (<1%). The odds of treatment interruption in people living with HIV were greater for clients with TB present than those with no TB. Treatment interruption was also more likely among clients receiving ART in urban clinics than rural, clients who were younger than 15 years old, and female clients. Although the factors playing a protective role in treatment interruption were not the direct focus of the current study, it appears that adjustments, such as MMD, made during the pandemic assisted people living with HIV to help lessen the burden caused indirectly by other factors. In future studies, analyzing those protective factors may be imperative, particularly through practice-based qualitative research.

## Figures and Tables

**Table 1:** Descriptive statistics by retention status for patients in HIV/AIDS clinics of Haut-Katanga and Kinshasa provinces, DRC, who initiated ART between 1 April 2019 and 30 March 2021.

Demographic and Clinical Characteristics	Frequency (%)	Outcome	
		Retained in Care	LTFU or Died
Interruption in treatment			
In care or transferred out	36,088 (99.1)		
Treatment interrupted	330 (0.9)		
ART initiation			
Pre-COVID-19	19,213 (52.5)	98.5%	1.5%
During COVID-19	17,372 (47.5)	99.8%	0.2%
Urban/rural status of the health zone			
Urban	27,255 (74.5)	98.8%	1.2%
Rural	9330 (25.5)	99.8%	0.2%
Patient’s sex			
Male	14,026 (39.1)	99.3%	0.7%
Female	21,833 (60.9)	98.9%	1.1%
Patient’s age at the time of last visit			
<15 years of age	2713 (7.4)	98.6%	1.4%
15 years or older	33,872 (92.6)	99.1%	0.9%
Age and sex			
<15 years, male	1201 (3.3)	99.0%	1.0%
<15 years, female	1440 (4)	98.2%	1.8%
15–49.9 years, male	9990 (27.9)	99.3%	0.7%
15–49.9 years, female	17,592 (49.1)	99.0%	1.0%
50 years or older, male	2830 (7.9)	99.3%	0.7%
50 years or older, female	2800 (7.8)	98.9%	1.1%
TB status			
No TB	35,302 (98.9)	99.1%	0.9%
TB detected based on treatment or Rx	376 (1.1)	95.6%	4.4%
**Clinical Characteristics (Continuous variable)**	**Frequency**	**Mean (SD)**	
Duration on ART in months	36,585	9.5 (6.4)	11.3 (4.4)

Notes: ART initiation was classified as “pre-COVID-19” if it occurred during 1 April 2019 and 9 Apri1 2020; it was classified as “during COVID-19” if initiated during 10 April 2020 and 30 March 2021. *p*-value for the continuous variable–duration on ART was derived from the t-test. Rx refers to medical prescriptions. Abbreviations: TB, tuberculosis.

**Table 2:** Firth’s logistic regression of interruption in treatment in patients in HIV/AIDS clinics of Haut-Katanga and Kinshasa provinces, Democratic Republic of Congo, January 2014–May 2019.

Variable	AOR	95% CI for AOR	*p*-Value
ART initiation			
Pre-COVID-19	**12.5**	8.5–18.3	<0.001
During COVID-19 *			
Client’s sex			
Female	**1.5**	1.2–1.8	0.002
Male *			
Urban/Rural status of the health zone			
Urban	**6.5**	3.9–10.9	<0.001
Rural *			
Age at the time of the last visit			
<15 years of age	**1.8**	1.3–2.5	0.001
15 years or older *			
TB status at the last visit			
TB detected based on treatment or Rx	**5.1**	3.0–8.5	<0.001
No TB detected *			
Duration on ART in months (continuous variable)	**0.9**	0.92–0.96	<0.001

Notes: * Indicates the reference category. The AORs in bold font indicate statistical significance of association at *p* < 0.05. Rx refers to medical prescription. Abbreviations: AOR, adjusted odds ratios; TB, tuberculosis.

## Data Availability

The program-implementing partners required that data be destroyed after publication. The authors have the data until the publication of the article. The authors can facilitate data access if requested with proper permission from the DRC Ministry of Health.
